# Reduction of polyethylenimine-coated iron oxide nanoparticles induced autophagy and cytotoxicity by lactosylation

**DOI:** 10.1093/rb/rbw023

**Published:** 2016-06-12

**Authors:** Jiuju Du, Wencheng Zhu, Li Yang, Changqiang Wu, Bingbing Lin, Jun Wu, Rongrong Jin, Taipeng Shen, Hua Ai

**Affiliations:** ^1^National Engineering Research Center for Biomaterials; ^2^Department of Radiology, West China Hospital, Sichuan University, Chengdu, 610065, P.R. China; ^3^Institute of Biochemistry and Cell Biology, Shanghai Institutes for Biological Sciences, Chinese Academy of Sciences, Shanghai, 200031, P. R. China and; ^4^School of Medical Imaging, North Sichuan Medical College, Nanchong, 637000, P.R. China

**Keywords:** SPIO, polyethyleneimine, lactosylation, MRI, RAW 264.7, autophagy

## Abstract

Superparamagnetic iron oxide (SPIO) nanoparticles are excellent magnetic resonance contrast agents and surface engineering can expand their applications. When covered with amphiphilic alkyl-polyethyleneimine (PEI), the modified SPIO nanoparticles can be used as MRI visible gene/drug delivery carriers and cell tracking probes. However, the positively charged amines of PEI can also cause cytotoxicity and restricts their further applications. In this study, we used lactose to modify amphiphilic low molecular weight polyethylenimine (C_12_-PEI_2K_) at different lactosylation degree. It was found that the N-alkyl-PEI-lactobionic acid wrapped SPIO nanocomposites show better cell viability without compromising their labelling efficacy as well as MR imaging capability in RAW 264.7 cells, comparing to the unsubstituted ones. Besides, we found the PEI induced cell autophagy can be reduced *via* lactose modification, indicating the increased cell viability might rely on down-regulating autophagy. Thus, our findings provide a new approach to overcome the toxicity of PEI wrapped SPIO nanocomposites by lactose modification.

## Introduction

Superparamagnetic iron oxide (SPIO) nanoparticles have been widely used in magnetic resonance imaging (MRI) owing to their unique properties in shorting spin-spin (*T_2_*) relaxation time of protons of water molecules. In the last decade, numerous studies have concentrated on the surface engineering of SPIO nanoparticles which can functionalize or improve them in many aspects, such as drug delivery, gene delivery and cell tracking [1–3]. Polyethylenimine (PEI) is one of the most commonly used polycations for gene delivery [4, 5], and can be used for forming of polycation/SPIO nanocomposites [6–8]. Importantly, the alkylation of PEI_25K_ is capable of phase transferring of hydrophobic SPIO nanoparticles into water and improving the *T_2_* relaxivity [9]. High-molecular weight PEI shows high transfection efficiency but accompanied by high cytotoxicity compared with low-molecular weight PEI due to higher number of amine groups on the polymer [4, 10]. Thus, in our previous work, we chose low-molecular-weight amphiphilic alkylated PEI of 2000 Da molecular weight (N-Alkyl-PEI_2K_) to wrap multiple SPIO nanoparticles with improved *T_2_* relaxivity. The N-Alkyl-PEI_2K_/SPIO nanocomposites have demonstrated reasonable applications in gene delivery [11, 12], cell labelling and *in vivo* tracking (such as mesenchymal stem cells [13], chondrocytes [14] and dendritic cells [15, 16]). However, the N-Alkyl-PEI_2K_/SPIO nanocomposites still have certain degree of cytotoxicity when used at higher concentrations.

In recent years, it has been demonstrated that nanoparticles can activate autophagy and leads to nanoparticle-induced toxicity [17–19]. Autophagy is a dynamic process of intracellular degradation with several sequential steps: (i) initiation of the phagophore; (ii) formation of autophagosome; (iii) fusion of autophagosome with lysosome, a.k.a. formation of autolysosome; (iv) utilization of degradation products by lysosomal enzymes [20, 21]. Autophagy is considered as a strategy for cell survival under stress, while excessive autophagy will lead to cell death. The machnisms of PEI induced cytotoxicity have been investigated in several studies previously [10, 22–24]. And recently, more detailed findings have revealed that the PEI-induced cytotoxicity is an autophagy event in a stepwise manner [25]. To better utilize PEI/SPIO nanocomposites in biomedical applications, one needs to overcome PEI-induced cytotoxicity. Therefore, we hypothesized that appropriate modification of PEI may reduce autophagy and result in improved cell viability.

Herein, we chose the previously well-studied N-alkyl-PEI (C_12_-PEI_2K_) as a model polycation and modified it with lactose at different lactosylation degree (6.8 and 11.7%). Then we used these polymers to encapsulate multiple hydrophobic SPIO nanoparticles, and studied if lactosylation of PEI can reduce its cytotoxicity *via* modulating autophagy. Furthermore, we tested how the lactosylation of PEI would affect its cell labelling efficacy and *T_2_* relaxivity.

## Materials and methods

### Materials

Mouse macrophage cell line RAW 264.7 was obtained from West China School of Pharmacy Sichuan University (Chengdu, China). RPMI-1640, phosphate buffered saline (PBS) and penicillin/streptomycin was purchased from Hyclone (USA). Fetal bovine serum (FBS) was purchased from Gibco (USA). Cell counting kit-8 (CCK-8) was purchased from Beyotime Institute of Biotechnology (Jiangsu, China). Mammalian Cell Lysis Reagent was purchased from Fermentas (USA). β-Actin antibody, P62 antibody, goat-anti-mouse-IgG-HRP and goat-anti-rabbit-IgG-HRP were purchased from Santa Cruz (USA). LC3 antibody was purchased from NOVUS (USA). Enhanced chemiluminescence (ECL) kit and PVDF membrane were purchased from Bio-Rad (USA). LPS (lipopolysaccharide) was purchased from Hycult Biotech (Netherlands).

### Preparation and characterization of SPIO-loaded C_12_-PEI_2K_-LAC nanoparticles (N-alkyl-PEI-LAC/SPIO)

Alkylated polyethyleneimine (PEI) of 2000 Da molecular weight (C_12_-PEI_2K_) was synthesized following a published protocol [9, 26]. PEI_2K_ was reacted with 1-iodododecane in ethanol. C_12_-PEI_2K_ was obtained as gummy solid by freeze-drying and was confirmed by ^1^H NMR (CDCl_3_) and Elemental analysis.

C_12_-PEI_2K_ was dissolved in water and added with lactobionic acid (LAC) [27]. The solution was adjusted to pH = 5 with diluted hydrochloric acid and added with 1-(3-Dimethylaminopropyl)-3-ethylcarbodiimide Hydrochloride. The mixture was stirred for 3 days at room temperature, then dialyzed in water and lyophilized to obtain C_12_-PEI_2K_-LAC. The product was confirmed by ^1^H NMR dimethylsulfoxide (DMSO) and Elemental analysis. Different grafting ratio of C_12_-PEI_2K_-LAC could be prepared by changing the ratio between C_12_-PEI_2K_ and LAC.

SPIO nanoparticles were synthesized following a method from Sun *et al.* [28]. SPIO nanoparticles were stored in hexane and dried by argon gas, and then dispersed in chloroform. C_12_-PEI_2K_-LAC was dissolved in DMSO, and then added into chloroform under sonication. A mixture of SPIO nanoparticle/C_12_-PEI_2K_-LAC at a mass ratio of 1: 0.6 was added into water under sonication. Chloroform and DMSO was stepwise removed by evaporation and dialysis. Size distribution and zeta potential of C_12_-PEI_2K_-LAC/SPIO nanocomposites was characterized through dynamic light scattering (DLS) and scanning electron microscope (SEM). Iron concentration of C_12_-PEI_2K_-LAC/SPIO nanocomposites solution was analyzed by atomic absorption spectroscopy.

### Cell culture

RAW 264.7 cells were cultured in RPMI-1640 medium containing 10% FBS, 100 U/ml penicillin, and 100 μg/ml streptomycin in a humidified atmosphere of 5% CO_2_ at 37°C with the medium changed every other day.

### Cell viability evaluation

CCK-8 was used to assess the cytotoxicity of nanocomposites. Cells were seeded in 96-well plates (3 × 10^3^ cells/well) and the nanocomposites were added in the following day and incubated for 24 h. CCK-8 solution (10 μl) was added to each well of the plate and incubated for 2 h in a CO_2_ incubator. The absorbance was measured at 450 nm using a microplate reader (Thermo Scientific, USA).

### Western blotting assay

RAW 264.7 cells were seeded in six-well plates at a density of 3 × 10^5^ cells/well, and incubated with indicated nanocomposites for 24 h in a CO_2_ incubator. The cells were harvested and lysed in Mammalian Cell Lysis Reagent and centrifuged at 13 000 rpm for 15 min at 4°C. Proteins from each sample were separated on a 12% SDS/PAGE gel and transferred to PVDF membrane (Bio-Rad, USA). After blocking with 5% nonfat milk in TBST at room temperature for 1 h, membranes were washed three times with TBST and incubated overnight at 4°C with primary mouse monoclonal antibody (β-Actin antibody, P62 antibody) and primary rabbit antibody LC3 antibody. The membranes were washed three times with TBST, followed by 1 h incubation at room temperature with goat anti-mouse-IgG-HRP and goat-anti-rabbit-IgG-HRP. After incubation, membranes were washed three times in TBST. Then the antigen-antibody complexes were visualized with ECL kit.

### GFP-LC3 dot formation assay

RAW 264.7 cells stably expressing GFP-LC3 were cultured on glass slides in a 24-well plate. Following treatment, cells were fixed with 4% paraformaldehyde for 15 min. The fixed cells were rinsed in PBS twice and mounted in Diamond Antifade Mountant (Life Technologies, USA). The images were observed and captured under a confocal laser scanning microscope (CLSM: TCS SP4, Leica Microsystems, Germany).

### Cellular uptake of nanocomposites

Intracellular iron content was detected by colorimetric ferrozine assay. RAW 264.7 cells were labelled with different concentrations of SPIO nanocomposites (2.5, 5, 10, 15 μg/ml) within 48-well culture plate for 24 h. Following three washes, iron-releasing reagent (mixture of equal volume of 1.4 M HCl and 4.5% (w/v) KMnO_4_ in H_2_O) was added and incubated for 2 h at 60°C within a drying oven. Upon cooled to room temperature, iron-detection reagent (6.5 mM ferrozine, 6.5 mM neocuproine, 2.5 M ammonium acetate and 1 M ascorbic acid dissolved in water) was added. Absorbance of the samples was read at 550 nm on a plate reader (Varioskan Flash, Thermo Scientific, USA). Iron contents were determined by matching the absorbance to those from a range of standard concentrations.

### MRI study of labelled cells

RAW 264.7 cells were labelled with nanocomposites for 24 h at 5 μg/ml (iron concentration). Cells were collected and washed with PBS twice, then fixed with 600 μl 4% paraformaldehyde for 30 min at 4°C. Cells were washed with PBS and mixed with 5% gelatin crosslinked with glutaraldehyde inside 250 μl microcentrifuge tubes. MRIwas performed under a clinical 3 T scanner (Achieva, Philips) and with a head coil. Axial images of the phantoms were acquired by *T*_2_-weighted spin echo sequence (TR = 5000 ms, TE = 6.90enc ms; number of averages, 1, matrix = 384 × 224, field of vision = 250 × 190 mm, slice thickness = 2.0 mm). Signal Intensities at different TE were measured to calculate the *T*_2_ value of each phantom.

### Statistical analysis

The results are presented as mean ±  SDs with *n* = 3, Statistical analysis was performed using a two-tailed Student’s *t* test. The differences were considered statistically significant with *P* values of *P < 0.05, **P < 0.01, and ***P < 0.001.

## Results and discussion

### Characterization of C_12_-PEI_2K_-LAC/SPIO nanocomposites

**Figure 1.**
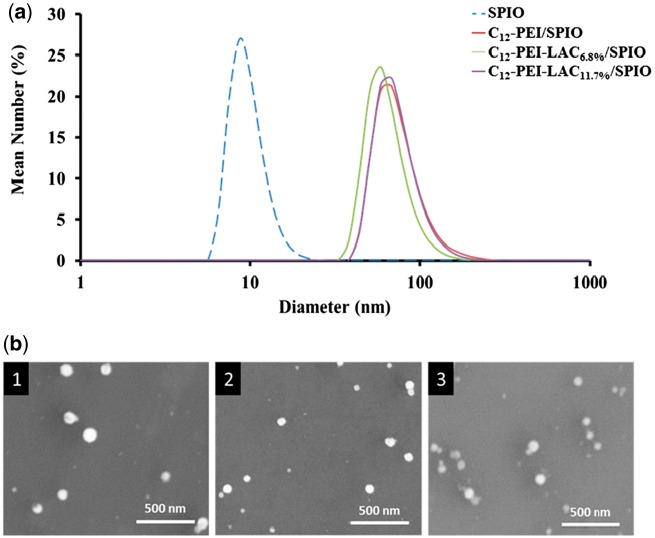
(a) DLS of SPIO, C_12_-PEI_2K_-LAC/SPIO nanoparticles. SPIO shows a diameter of 9.0 ± 0.6 nm in hexane, C_12_-PEI_2K_/SPIO shows a diameter of 73 ± 5 nm, C_12_-PEI_2K_-LAC_6.8%_/SPIO shows a diameter of 67 ± 4 nm, C_12_-PEI_2K_-LAC_11.7%_/SPIO shows a diameter of 75 ± 3 nm. **(b)** SEM of C_12_-PEI_2K_/SPIO (1), C_12_-PEI_2K_-LAC_6.8%_/SPIO (2), and C_12_-PEI_2K_-LAC_11.7%_/SPIO (3).

C_12_-PEI_2K_ and C_12_-PEI_2K_-LAC were synthesized following published protocols [9, 26, 28] and confirmed by ^1^H NMR spectrum. C_12_-PEI_2K_ (400 MHz, CDCl_3_): δ 2.97–2.30 (–NH–*CH_2_–CH_2_*–NH–), 1.54–1.16 (*–CH_2_–(CH_2_)*_10_CH_3_), 0.87 (–CH_2_–(CH_2_)_10_*CH_3_*); C_12_-PEI_2K_-LAC (400 MHz, DMSO): δ 4.74–3.12 (LAC), 3.11-2.30 (–NH–*CH_2_–CH_2_*–NH–), 1.34-1.07 (–CH_2_–(CH_2_)_10_*CH_3_*), 0.85 (–CH_2_–(CH_2_)_10_*CH_3_*). The LAC grafting ratio of C_12_-PEI_2K_-LAC was calculated by the changes of carbon (C) and nitrogen (N) contents. Elemental analysis shows the C/N ratio of C_12_-PEI_2K_ is 3.54, and the C/N ratio of C_12_-PEI_2K_-LAC is 4.36 and 4.94. So ratio of Sugar unit’s C element to N of polymer is 0.82 and 1.40. Sugar unit have 12 C, and respectively the grafting ratio is 6.8% and 11.7. And Figure 1 shows that the SPIO have a relatively narrow size distribution in hexane, and have a mean diameter of 9.0 ± 0.6 nm characterized by DLS. C_12_-PEI_2K_/SPIO shows a diameter of 73 ± 5 nm, C_12_-PEI_2K_-LAC_6.8%_/SPIO shows a diameter of 67 ± 4 nm, C_12_-PEI_2K_-LAC_11.7%_/SPIO shows a diameter of 75 ± 3 nm in water.

### Cell viability evaluation of C_12_-PEI-LAC/SPIO nanocomposites

**Figure 2.**
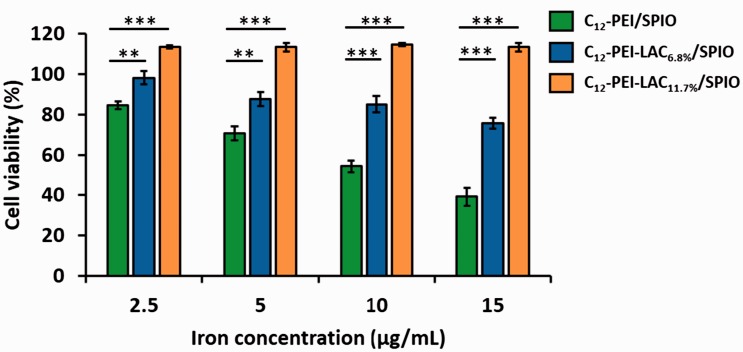
Cell viability of C_12_-PEI/SPIO, C_12_-PEI-LAC_6.8%_/SPIO, C_12_-PEI-LAC_11.7%_/SPIO in RAW 264.7 cells was evaluated by CCK-8 assay. Results were expressed as the mean ± SD, *n* = 3; *(*P* < 0.05), **(*P* < 0.01), ***(*P* < 0.001) vs. C_12_-PEI/SPIO alone group.

The transfection efficiency of PEI is accompanied with certain degrees of cytotoxicity, depending on its molecular weight. Previously, a number of studies have demonstrated that low molecular weight PEI shows satisfactory transfection and minor cytotoxicity [29, 30]. To understand how lactose modification of PEI would affect the SPIO loaded nanocomposites, we assessed the cell viability after incubation with different concentrations of nanocomposites for 24 h. A standard CCK-8 assay was used to evaluate the cytotoxicity of nanocomposites in RAW 264.7 cells. As shown in Figure 2, two series of C_12_-PEI-LAC/SPIO nanocomppsites showed higher cell viability compared with C_12_-PEI/SPIO nanocomposites. Besides, with the higher degree of lactosylation, the cell viability was markedly improved. Especially, when the iron concentration is 15 μg/ml, the C_12_-PEI-LAC_11.7%_/SPIO group showed no obvious cytotoxicity, while the C_12_-PEI/SPIO group had only ∼40% cell viability. These results demonstrated that the lactose modification can considerably reduce the PEI-induced cell death.

### Reduction of PEI-induced autophagy in RAW 264.7 cells through lactosylation

**Figure 3.**
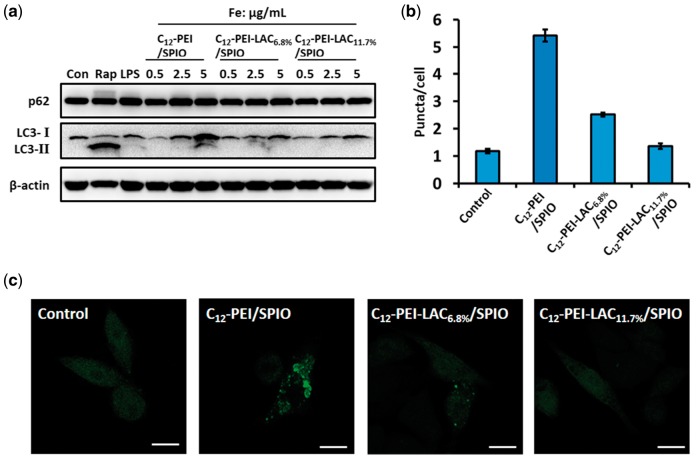
C_12_-PEI/SPIO nanocomposites induced autophagy can be prevented by PEI lactosylation. **(a)** Western blotting assay of RAW 264.7 cells that were untreated (negative control), treated with rapamycin (50 μg/ml, 8 h), LPS (1 μg/ml 24 h) and nanocomposites (0.5, 2.5, 5 μg/ml, 24 h). Both rapamycin and LPS groups served as positive controls. The RAW 264.7 cells stably expressing GFP-LC3 were labelled with nanocomposites at an iron concentration of 5 μg/ml for 24 h. **(b)** the GFP puncta in RAW 264.7 cells were measured and summarized. Data were the mean value of three independent experiments with each count of no less than 60 cells. Values are expressed as the mean ± SD, *n* = 3. **(c)** the representative images of GFP puncta in RAW 264.7 cells after treatment under a CLSM. Scale bar: 10 μm.

PEI can induce autophagy which is considered as a major cause of cytotoxicity besides apoptosis [25]. To determine how lactosylation of PEI would affect autophagy, we detected the autophagy flux in RAW 264.7 cells after the treatment with nanocomposites. First, we investigated LC3 conversion (from LC3-I to LC3-II) and p62 degradation by immunoblotting, as they are two well-established markers of autophagy [31, 32]. And we found that in C_12_-PEI/SPIO nanocomposites treated cells, LC3-I converted to LC3-II with a dose-dependent manner, while in C_12_-PEI-LAC/SPIO nanocomposites groups showed no obvious LC3-II formation (Fig. 3a). And rapamycin (Rap) and LPS served as positive controls which can induce autophagy through different mechanisms [33–35]. Interestingly, the PEI induced autophagy did not lead to degradation of p62 (Fig. 3a), which might indicate the positive amine groups of PEI can elevate lysosomal pH and result in insufficient protein degradation. But it is also possible that PEI activates autophagy through Toll-like receptor 4 which leads to up-regulation of p62 in a non-classical autophagy pathway [36, 37]. Because in C_12_-PEI/SPIO group, when Fe concentration is 2.5 or 5 μg/ml, p62 showed a slight increase similar to LPS group.

To further confirm the protective role of lactosylation in autophagy induction, we used a stable RAW 264.7 cell line expressing GFP-LC3 to observe the autophagosome formation. After 24 h incubation with different nanocomposites, the GFP-LC3 puncta were observed and captured under a CLSM. As shown in Figure 3b and c, the numbers of green fluorescent dots were increased after treated with C_12_-PEI/SPIO nanocomposites, compared with the untreated group (control). However, the lactosylation group showed a dramatic decrease in autophagosome formation which is close to the untreated group. Taken together, these results demonstrated that lactosylation of PEI can reduce its induced autophagy which contributes to the improved cell viability.

### The effects of lactosylation on surface charge and intracellular uptake of C_12_-PEI/SPIO nanocomposites

**Figure 4.**
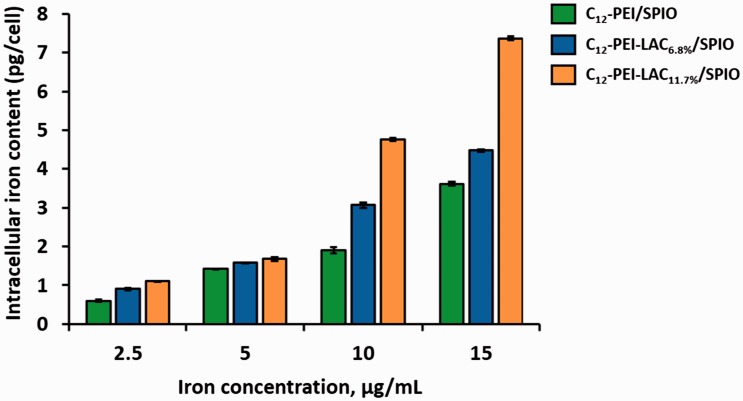
The iron content of RAW 264.7 cells after labelled with SPIO-loaded nanocomposites for 24 h at varied iron concentrations.

Our results showed that the lactosylation of PEI can reduce autophagy and lead to the reduced cytotoxicity. To unveil how lactose modification can down regulate autophagy, we first analyzed the surface charge of nanocomposites. C_12_-PEI/SPIO nanocomposites are positively charged with zeta potentials of +34.6 mV, which is higher than C_12_-PEI-LAC/SPIO nanocomposites. The zeta potentials of C_12_-PEI-LAC_6.8%_/SPIO is +33.4 mV and C_12_-PEI-LAC_11.7%_/SPIO is +28.7 mV. Therefore, we concluded that lactosylation can partially shield the positive charge of PEI. It is possible that with the decreased surface charge, cellular uptake of nanoparticles is also reduced. It has been reported that the positive surface charge of nanoparticles is positively correlated with cellular uptake [38]. Cellular iron content was measured to determine the internalization degree of nanocomposites. Different from previous reports, the C_12_-PEI-LAC/SPIO nanocomposites showed an increased cellular uptake, particularly at high iron concentrations (Fig. 4). Briefly, our data indicated that lactose modification of PEI can decrease its positive charge and may promote C_12_-PEI-LAC/SPIO cellular uptake by macrophages at high iron concentrations.

### MRI study of C_12_-PEI-LAC/SPIO nanocomposites in cell labelling

**Figure 5.**
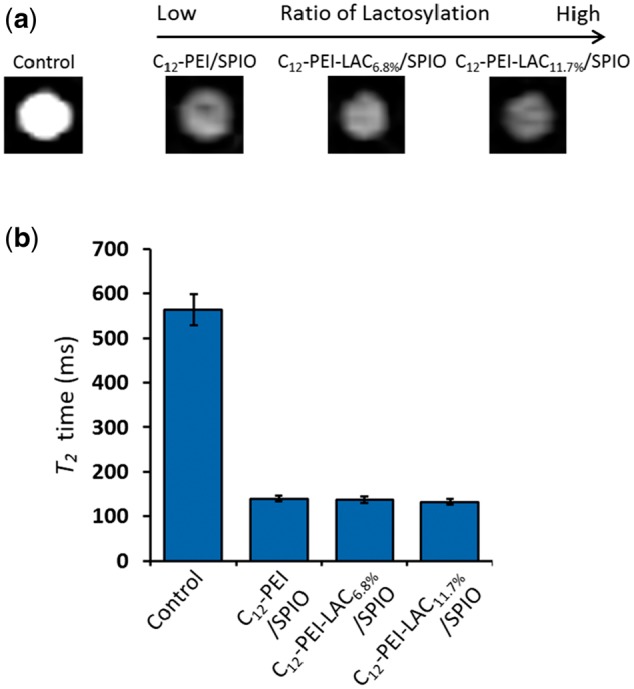
*In vitro* MRI study of SPIO-loaded nanocomposites (5 μg/ml) labelled RAW 264.7 cells for 24 h. **(a)** *T*_2_ values of labelled RAW 264.7 cells as a function of ratio of lactosylation. **(b)** Definition of the detectable threshold of SPIO-labelled RAW 264.7 cells in gelatin phantoms.

SPIO-based MRI contrast agents could shorten the *T*_2_ (spin-spin) relaxation time. To figure out whether the lactosylation of PEI will affect its capability as a high efficient MRI contrast agent, we studied the MR imaging of C_12_-PEI-LAC/SPIO nanocomposites labelled cells. We first labelled RAW 264.7 cells with unmodified and modified PEI wrapped SPIO nanoparticles for 24 h at the same iron concentration (5 μg/ml). And under a clinical 3 T scanner, we performed the *in vitro* MR imaging study of SPIO-laden cells. Under *T*_2_ scan, *T*_2_ value decreased from ∼560 to ∼150 ms with the SPIO loaded nanocomposties labelling (Fig. 5). In addition to that, it is noteworthy that lactosylation of PEI did not show any dramatic difference in *T*_2_ value, comparing to C_12_-PEI/SPIO nanocomposites. Thus these results demonstrated that latosylation of PEI is able to improve cell viability without compromising its capability in shortening *T*_2_ relaxation times.

**Scheme 1.**
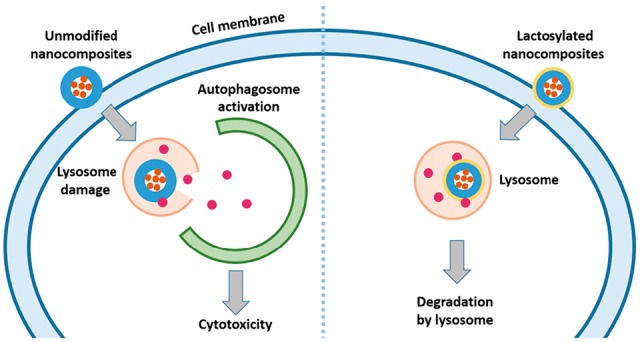
The suggested model of how PEI lactosylation prevents autophagy and improves cell viability.

## Conclusions

To utilize PEI wrapped SPIO nanocomposites for tracing cells of interest *in vivo* under clinic MR scanners, the cytotoxicity is still a major concern. In this study, we used an N-alkyl-PEI (C_12_-PEI_2K_) as a model polycation to test if the lactose modification of PEI can reduce its induced cytotoxicity. Our data showed that lactosylation significantly decreased PEI-induced cytotoxicity. And further investigations demonstrated that lactosylaltion can down regulate autophagy which contributes to the improved cell viability. As suggested in Scheme 1, we propose that C_12_-PEI/SPIO nanocoposites could induce lysosome damage (or lysosome dysfunction at low dose) followed by autophagy activation which leads to cytotoxicity, while with the surface decoration of lactose, C_12_-PEI-LAC/SPIO nanocomposites can be degraded by lysosome gradually without eliciting obvious cytotoxicity. More importantly, we showed that the lactosylation did not affect cellular uptake of nanocomposites and resulted in an uncompromised *T*_2_ relaxivity. In conclusion, we provide a unique approach to overcome the PEI wrapped SPIO nanoparticles induced cytotoxicity by down-regulating autophagy and this modification will not affect its efficiency in MR imaging as contact agents.

## Funding

This work was supported by grants from National Key Basic Research Program of China (2013CB933903), National Key Technology R&D Program (2012BAI23B08), and National Natural Science Foundation of China (20974065, 51173117 and 50830107).

*Conflict of interest statement*. None declared.
